# Social synchronization of circadian rhythms with a focus on honeybees

**DOI:** 10.1098/rstb.2020.0342

**Published:** 2021-10-11

**Authors:** Oliver Siehler, Shuo Wang, Guy Bloch

**Affiliations:** ^1^ Department of Ecology, Evolution and Behavior, The Alexander Silberman Institute of Life Sciences, The Hebrew University of Jerusalem, Givat-Ram, Jerusalem 91904, Israel; ^2^ The Federmann Center for the Study of Rationality, The Hebrew University of Jerusalem, Givat-Ram, Jerusalem 91904, Israel; ^3^ Department of Mechanical and Aerospace Engineering, The University of Texas at Arlington, Arlington, TX 76010, USA

**Keywords:** social insects, honeybees, social entrainment, circadian rhythms, coupled oscillators, self-organization

## Abstract

Many animals benefit from synchronizing their daily activities with conspecifics. In this hybrid paper, we first review recent literature supporting and extending earlier evidence for a lack of clear relationship between the level of sociality and social entrainment of circadian rhythms. Social entrainment is specifically potent in social animals that live in constant environments in which some or all individuals do not experience the ambient day-night cycles. We next focus on highly social honeybees in which there is good evidence that social cues entrain the circadian clocks of nest bees and can override the influence of conflicting light-dark cycles. The current understanding of social synchronization in honeybees is consistent with self-organization models in which surrogates of forager activity, such as substrate-borne vibrations and colony volatiles, entrain the circadian clocks of bees dwelling in the dark cavity of the nest. Finally, we present original findings showing that social synchronization is effective even in an array of individually caged callow bees placed on the same substrate and is improved for bees in connected cages. These findings reveal remarkable sensitivity to social time-giving cues and show that bees with attenuated rhythms (weak oscillators) can nevertheless be socially synchronized to a common phase of activity.

This article is part of the theme issue ‘Synchrony and rhythm interaction: from the brain to behavioural ecology’.

## 1. Introduction

### (a) Circadian clocks

Many physiological and behavioural processes in diverse organisms including animals, plants, and some bacteria vary with rhythms of about a day. These rhythms are defined as endogenous ‘circadian’ if they meet the following three criteria: (i) they persist or ‘free run’, with a period of about (‘*circa*’ in Latin) 24 h (*'dien'* = a day) in the absence of external time-giving cues (known as *'zeitgebers*', for example, daily oscillations in light intensity or ambient temperature); (ii) their period length is stable in a wide range of physiologically relevant temperatures (known as ‘temperature compensation’); and (iii) the phase of these endogenous rhythms is determined (‘entrained’) by environmental cues [1]. Endogenous timekeeping mechanisms are thought to be functionally significant because they allow organisms to anticipate recurring changes in their environment, adjust their physiology and behaviour to their environment, and help coordinate internal processes [1–3]. Circadian rhythms are generated by endogenous clocks that function in many tissues and influence vital processes throughout the body. Perturbations to the molecular clock machinery, for example, because of mutations in clock genes or misalignment between endogenous and exogenous environmental cycles, are associated with numerous diseases (for recent reviews see [4,5]). For example, in modern human societies, many individuals experience frequent misalignment between endogenous and societal rhythms, creating a discrepancy between sleep timing on work/school days and work-free days. This phenomenon, which was termed ‘social jetlag’, is now known to be associated with many of the same metabolic, cardiovascular and psychiatric risks that have been found in shift workers or repeated jetlag owing to travel across time zones [6,7].

From an evolutionary perspective, circadian clocks are thought to have evolved as adaptations to geophysical cycles generated by the rotation of our planet around its axis [2,8] which generate strong fluctuations in exposure to sun radiation. Organisms that evolved an endogenous circadian clock could anticipate these environmental changes and organize cellular and physiological processes in a way that best fit these cycles (for example, protecting DNA replication from UV radiation; [9]). Along with this notion, the vast majority of studies on the entrainment of circadian rhythms have focused on photic entrainment which is considered evolutionarily ancient and the most important time cue to circadian clocks. Non-photic entrainment has received substantially less attention, but recently there has been significant progress in research on entrainment by temperature and feeding cycles (e.g. [10–14]). There also is good evidence that non-photic, non-thermal, cues may act as potent zeitgebers. These include social interactions between individuals that have long been known to entrain the circadian clock in various animal species (reviewed in [15–18]).

### (b) Social entrainment of circadian clocks

Synchronized activity rhythms of individuals of the same species may help coordinate their behaviours towards a common goal such as mating, care for offspring or common activities such as defence or social foraging [15,17,19–21]. Social synchronization of circadian rhythms can also enable segregating the activity of individuals such as in the case of subordinate males avoiding a dominant aggressive neighbour (sometimes termed ‘social desynchronization’; [19,22]). It should be noted that *social synchronization* of daily activity rhythm does not necessarily imply that the circadian clock is *socially entrained*. In order to confirm social entrainment, it is necessary to show that social cues altered the phase or free-running period of the circadian clock, and that this phase is retained after the animal is deprived of the social cues. Social synchronization is thus potentially important to many animals because it can help coordinate activities such as courtship and parental care, but predicted to be specifically important for social animals that coordinate many aspects of their daily life. Reviews of the literature, however, provide little support for this notion (table 1; [17]). For example, there is good evidence for social entrainment in animals that are not considered social, such as fruit flies [23,40], whereas social species such as the Mongolian gerbil or sugar glider failed to be entrained by even strong and ecologically relevant interactions such as aggression or mating (reviewed in [17,18]). Table 1 provides an updated list of studies on social entrainment in relation to their social lifestyle.

**Table 1.** Studies testing social entrainment of circadian rhythms in animals.

animal species	order/class	degree of sociality	social interactions	entrainment	references
fruit fly *(Drosophila melanogaster)*	Diptera, Insecta	solitary to facultative gregarious	contact with other males	fair	[23]
			sexual contact with a female	no	[20,24]
			sexual contact with a female	weak	[25]
Madeira cockroach *(Leucophaea maderae)*	Dictyoptera, Insecta	gregarious	contact with conspecifics	no	[26]
honeybee (*Apis mellifera)*	Hymenoptera, Insecta	highly eusocial	direct/indirect contact with conspecifics, volatiles from hive	very good	[27,28]
glowworm *(Arachnocampa tasmaniensis*)	Diptera, Insecta	gregarious	bioluminesce of conspecifics	fair	[29]
glowworm *(Arachnocampa flava)*	Diptera, Insecta	gregarious	bioluminesce of conspecifics	no	[29]
sugar glider *(Petaurus breviceps)*	Marsupialia, Mammalia	social	contact with the opposite sex	no	[30]
rhesus monkey *(Macaca mulatta)*	Primates, Mammalia	social	contact with conspecifics	good	[31]
golden hamster (*Mesocricetus auratus*)	Rodents, Mammalia	solitary	various assays	no to fair	reviewed in [17,32]
Indian palm squirrel *(Funambulu spalmarum)*	Rodents, Mammalia	pair to group living	contact with other males	fair	[33]
Mongolian gerbil *(Meriones unguiculatus)*	Rodents, Mammalia	social	acoustic and olfactory communication	no	[34]
common marmoset (*Callithrix jacchus)*	Primates, Mammalia	social	acoustic communication	weak	[35]
			cohabitation	weak	[36]
leschenaults rousettte (*Rousettus leschenaultia)*	Chiroptera, Mammalia	gregarious to social	acoustic communication	good	[37]
Schneider's leaf-nosed bat *(Hipposideros speoris)*	Chiroptera, Mammalia	gregarious to social	indirect contact	good	[38]
grass rats (*Arvicanthis niloticus)*	Rodents, Mammalia	social	direct contact	no	[39]

Bats and bees currently provide the best evidence for social synchronization. In these social cavity dwelling species, individuals that experience the outside environment socially entrain the circadian clocks of colony-mates that do not leave the dark and temperature-stable cavity. The importance of cavity dwelling for the evolution of social entrainment is further supported by a recent study comparing two related forests and facultative cave *Arachnocampa* glowworms [29]. Glowworms are gregarious fly larvae that produce light (bioluminescence) to attract prey to their webs. Berry *et al*. [29] provide evidence that individual larvae of cave-dwelling *Arachnocampa tasmaniensis* are socially entrained by the bioluminescence of conspecifics inhabiting their caves. On the other hand, the related forest living *Arachnocampa flava* larvae are not synchronized to each other, but are rather entrained to the same ambient light : dark (LD) cycle. The evidence that life in relatively arrhythmic environments is associated with potent social entrainment is important because the dark, temperature-stable habitable zone is arguably the largest portion of the entire biosphere [41]. This includes habitats such as polar regions (over several months), the deep-sea, subterranean habitats, as well as caves and other cavities. Thus, social synchronization of circadian rhythms is potentially far more important than is currently appreciated.

Little is known about the neurobiology underlying social synchronization. The social cues and sensory modalities mediating social entrainment are diverse and depend on the social system and biology of the studied species. For example, relatively weak photic signals produced by bioluminescence appear to mediate social entrainment in the glowworm *A. tasmaniensis* [29]. In bats (table 1) and some passerine birds [42,43], there is evidence that species-specific vocalization can entrain circadian rhythms, suggesting that acoustic signals mediate social entrainment in these species. Olfactory cues seem to mediate social synchronization in honeybees (see below) and in some species of rodents (e.g. [44]). The clock input pathways mediating social entrainment are probably best understood in the fruit fly *Drosophila melanogaster* in which studies using mutant and transgenic lines convincingly showed that social entrainment is mediated by volatile pheromones and detected by the olfactory system [23,45,46]. There is also evidence that the DN1 pacemaker neurons that are part of the brain circadian network are influenced by social time cues and may be the cells relaying neuronally encoded social information to the clock [25,47].

The brief updated review above shows that we are just beginning to understand the mechanisms, generality and functional significance, of social synchronization. The insights suggested by the available literature are based on studies with relatively few species and need to be substantiated by careful studies of additional species representing diverse social lifestyles and habitats. In the following sections, we focus on studies on the Western honeybee *Apis mellifera* for which the functional significance and sociobiology of social synchronization are currently best understood.

### (c) Social synchronization in honeybees is potent, can override photic entrainment and does not require direct contact

Early studies established that groups of bees that are each entrained to a different phase, merge into a common activity phase after a couple of days of cohabitation [48]. However, given that in these experiments, the phase was recorded for groups of bees; these studies could not uncouple social masking (i.e. influences of social interactions on activity which are not mediated by circadian clocks) from genuine entrainment of the endogenous circadian clock. Recently, Fuchikawa *et al*. [27] used a system in which the circadian phase is determined for individually isolated bees after removing them from the social environment, and thus uncoupling masking and entrainment. Using this system, the authors showed that newly emerged workers experiencing the colony environment for the first 2 days (but not only the first day) post-pupal emergence show strong entrainment to the colony phase. They further tested workers in colonies experiencing conflicting phases of foraging activity (social time cues) and light/dark illumination regime. They discovered that the circadian phase of the focal young bees was similar to that of foragers and not aligned with the illumination regime. Similar results were obtained when the nest bees were confined to mesh enclosures in the centre of the nest, preventing them from visiting the hive entrance or periphery in which they could experience zeitgebers such as sunlight or ambient temperature. These experiments provide the first evidence that social cues can be more powerful than photic cues in entraining the circadian clock of an animal. The premise that social cues provide a stronger Zeitgeber than the LD cycle is supported by an independent study using a set-up that simultaneously measures the temperature of a mini queenless colony and the locomotor activity rhythms of individually caged workers with contact to the mini colony [28].

The social environment of a honeybee colony is rich and complex and many social cues and signals can potentially mediate social synchronization [49]. Early suggestions that the queen entrains the phase of workers in her colony [50] are not consistent with observations that queens are active around the clock with no circadian rhythms ([51–53]; T. Gernat, S. Silverstein-Krim, G.E. Robinson and G. Bloch 2015, unpublished observations). Furthermore, the queen is not likely to be entrained by the ambient environment; she shows negative phototaxis and spends most of her time in the centre of the hive which is dark and tightly thermoregulated [53]. The evidence that worker bees which are removed from the hive and monitored individually in constant conditions show circadian rhythms in locomotor activity similar to the hive phase, even if caged in the hive centre, indicates that they are not entrained by exposure to ambient time-givers [27,54–56]. The effective entrainment of bees that are caged in double-mesh enclosures further shows that direct contact with other bees is not necessary for social entrainment in honeybees. Thus, social network models which are based on direct contact between individuals do not seem to be a promising approach for explaining social synchronization in honeybee colonies. The available studies better fit self-organization models in which the sum activity of many individuals in a group are assembled into fluctuations in the microenvironment of the hive, which in turn entrain the circadian clocks of an increasing number of bees; the more individuals are entrained to the same phase, the stronger are the oscillations in the hive environment, and their capacity to entrain additional bees to the most common phase. Ultimately, these fluctuations are strong enough to entrain the whole colony [49]. The agreement with these models suggests that surrogates of worker activity mediate social synchronization in honeybee colonies and focuses the research on proxies of activity that can entrain the circadian clocks of honeybees.

### (d) What are the cues mediating social entrainment in honeybees?

The social environment influences many aspects of honeybee physiology including the strength and ontogeny of circadian rhythms and sleep (reviewed in [17,57]). Given the richness and complexity of the colony environment, it is challenging to identify the most important social zeitgebers. The double-mesh experiments described above indicate that direct contact with other bees is not necessary for social synchronization in honeybee colonies. Given that the hive cavity is dark and that temperature is a strong entraining cue in many animals, it is logical to suppose that temperature cycles may mediate social synchronization. Insect body temperature is typically elevated when active [58,59], and temperature cycles have been shown to entrain the circadian clock in several insect species (e.g. [60,61]). However, the brood area in which the young nurse bees spend most of their time is tightly thermoregulated, and in typical colonies is kept at 35 ± 0.5°C even under fluctuating ambient temperature conditions [62–64]. Furthermore, laboratory experiments showed that temperature oscillations with amplitudes of at least 6–10°C were needed to stably entrain bees [65,66]. Therefore, temperature cycles are not likely to mediate the social entrainment of circadian rhythms in honeybee colonies. Other surrogates of worker activity to consider include airborne and substrate-borne vibrations, volatile pheromones, hive odours, or gases such as CO_2_ or O_2_ which are influenced by bee metabolic activity [49]. Vibrations, volatile pheromones and hive odours are communication signals known to coordinate diverse activities in social insect colonies and are surrogates of activity and therefore good candidates for studies on the mechanism of social synchronization.

Already in 1994, Moritz and Kryger showed that allowing airflow between two groups of bees separated by a partition improved their synchronization to a common daily rhythm, lending credence to the premise that volatiles are important for social synchronization [48]. We have recently performed experiments in which we showed that both substrate-borne vibrations generated by forager activity, and volatiles drawn from a free-foraging colony stably entrain circadian rhythms in locomotor activity in small groups of young honeybees [67]. These experiments are consistent with the hypotheses that these two surrogates of activity mediate social entrainment in honeybee colonies. The specific volatile chemicals and vibratory cues are still to be determined. One possible volatile chemical that may play a role is CO_2_. The concentration of CO_2_ shows daily oscillations that are correlated with the foragers' morning departures and accumulated arrivals in the late afternoon, providing a surrogate to forager activity [68,69]. There are also oscillations in NO_2_, but not in O_2_ which are kept almost constant and similar to those of the external environment [69]. There is also some evidence that CO_2_ can entrain circadian rhythms in insects [70]. In mammals, it was shown that changes in CO_2_ concentration act at the cellular level and can phase shift oscillations in clock gene expression in cell culture [71]. CO_2_ may also entrain circadian rhythms indirectly by affecting worker activity. Honeybees regulate CO_2_ levels by fanning with their wings near the hive entrance, and there is a positive correlation between CO_2_ levels inside the hive and the number of fanning bees ([72]; reviewed in [73]). Thus, the increase in CO_2_ levels during the day may stimulate the activity of nest bees and entrain their clock. Airborne and substrate-borne vibrations are used in honeybee communication and facilitate coordinating colony-level activities (reviewed in [74,75]). Worker activity can generate substrate-borne vibrations in the honeycomb that are tightly correlated with the time of foraging activity. Moreover, there is evidence that substrate-borne vibrations entrain circadian rhythms in *D. melanogaster* [76].

Additional studies are necessary to establish that volatile signals and substrate-borne vibrations function as zeitgebers in freely foraging colonies and for identifying the specific chemicals and vibrational cues mediating social entrainment in honeybees. Taken together the studies with honeybees are consistent with the hypothesis that surrogates of the activity of workers with strong circadian rhythms (and specifically foragers) can create oscillations in the colony environment which in turn entrain the circadian clock of nest bees, including these with much weaker rhythms. However, it is not clear whether surrogates of activity can also effectively synchronize the activity rhythms of bees (such as callow bees) with weak or no circadian activity rhythms.

### (e) Social synchronization in groups of only young bees

Newly emerged bees (‘callows’) typically show attenuated or no circadian rhythms in locomotor activity and thus can be regarded as weak oscillators. Their low level of activity and weak (or absent) circadian rhythms cast doubt on whether synchronization by surrogates of their activity can effectively synchronize their circadian clocks. Nevertheless, studies in which young bees were monitored after being in small groups show that they are significantly better synchronized with each other compared to similar bees that were each kept individually isolated for a similar period. Their social synchronization was weaker compared to bees of a similar age removed from a free-foraging colony, even if they were isolated individually in the colony [27,77]. Our unpublished results suggest that synchronization may be somewhat better in groups of 100 compared to 30 callow bees (S. Silverstein-Krim and G. Bloch 2015, unpublished data). These studies suggest that social entrainment of circadian rhythms can occur even in relatively small groups of individuals with weak rhythms. It is, however, unknown if their circadian system is sufficiently sensitive to the social cues mediating social synchronization to support the social synchronization of callow bees, each caged in an individual cage.

In the following sections, we report an original experiment testing this question as well as starting to explore the mechanisms supporting social synchronization among bees with weak or no circadian rhythms in locomotor activity. We then discuss the implications of this experiment for our understanding of the social synchronization of circadian rhythms in honeybees. To assess phase synchronization we used, in addition to circular statistic, our recently developed pipeline for determining coupling strength for each pair of bees [78]. This pipeline, which we termed ‘inferring connections of networks' (ICON), is based on a unified data-driven graph-theoretic approach. It efficiently and reliably infers the dynamics of even complex networks of coupled oscillators and can be used with noisy data such as locomotor activity. We reasoned that the high sensitivity of the ICON procedure will enable us to study the dynamics of social synchronization among bees with attenuated circadian rhythms (weak oscillators). Even though the double-mesh separation experiments of Fuchikawa *et al*. [27] and Beer *et al*. [28] show that close-distance contact between individuals is not necessary for social entrainment in honeybees, it is still possible that direct contact improves social synchronization. Honeybees in a colony often antennate, touch and lick each other, and these close-contact interactions are important for colony coordination. Thus, we manipulated the contact between the bees by means of connecting adjacent cages with small tubes with a mesh separation that prevented moving from one cage to the other. Tube connection may also improve the propagation of volatiles and substrate-borne vibration that can entrain circadian rhythms in young bees ([67]; see above). We predicted that if cage contact improves social synchronization, then phase coherence in the circular statistics and coupling strength in the ICON analyses (see Material and methods below) will be higher for bees in connected compared to unconnected cages. We hypothesized that substrate-borne vibrations are important in this system and thus, predicted that coupling strength will be higher for bees placed on the same tray compared to bees at a similar distance but on a different tray. On the other hand, given that odours spread as a function of distance, we predicted that if olfactory signals mediate social synchronization, then coupling strength will be similar for bees on the same or on a different tray, as long as they are similarly distant from each other.

## 2. Material and methods

### (a) Bees

Honeybees were obtained from colonies maintained according to standard beekeeping techniques at the Bee Research Facility at the Edmond J. Safra campus of the Hebrew University of Jerusalem, Givat-Ram, Jerusalem, Israel. The bees represent a mixture of subspecies typical to Israel. To obtain newly emerged bees, we removed honeycomb frames with emerging worker pupae, brushed off all adult bees and immediately transferred each frame into a separate lightproof container. We placed the frames in an incubator (33 ± 1°C, 60 ± 5% relative humidity (RH)) for the bees to emerge. The emerging bees were collected from the comb within 2 h post-emergence under dim red light (DD) to avoid influences of light on their circadian system.

### (b) Monitoring locomotor activity

We placed each bee individually in a monitoring cage made of a modified Petri dish (diameter = 90 mm) provisioned with ad libitum sugar syrup (50% w/w) and pollen. The monitoring cages with the bees were placed in an environmental chamber (29 ± 1°C, 60 ± 5% RH). The chamber was illuminated with dim red light (Edison Federal EFEF 1AE1 Far (Cherry) Red LED; mean wavelength = 740 nm, maximum and minimum wavelengths were 750 and 730, respectively). Locomotor activity (measured as a number of pixels travelled over a time unit on the camera field of view and transformed to millimetres) was recorded automatically at a frequency of 1 Hz using the ClockLab data acquisition system (Actimetrics Inc., Evanston, IL, USA). The system is composed of four infrared light-sensitive black and white Panasonic WV-BP334, 0.08 lux CCD video cameras and a high-quality monochrome image acquisition board (IMAQ 1409, National Instruments). Each camera records the activity of 30 cages (each defined as an ‘arena’) that are placed on the same tray. In each trial, we monitored the movement of up to 116 bees; four additional cages, one on each tray, were left empty to provide records of background noise.

### (c) Analyses of circadian rhythms

**Figure 1.**
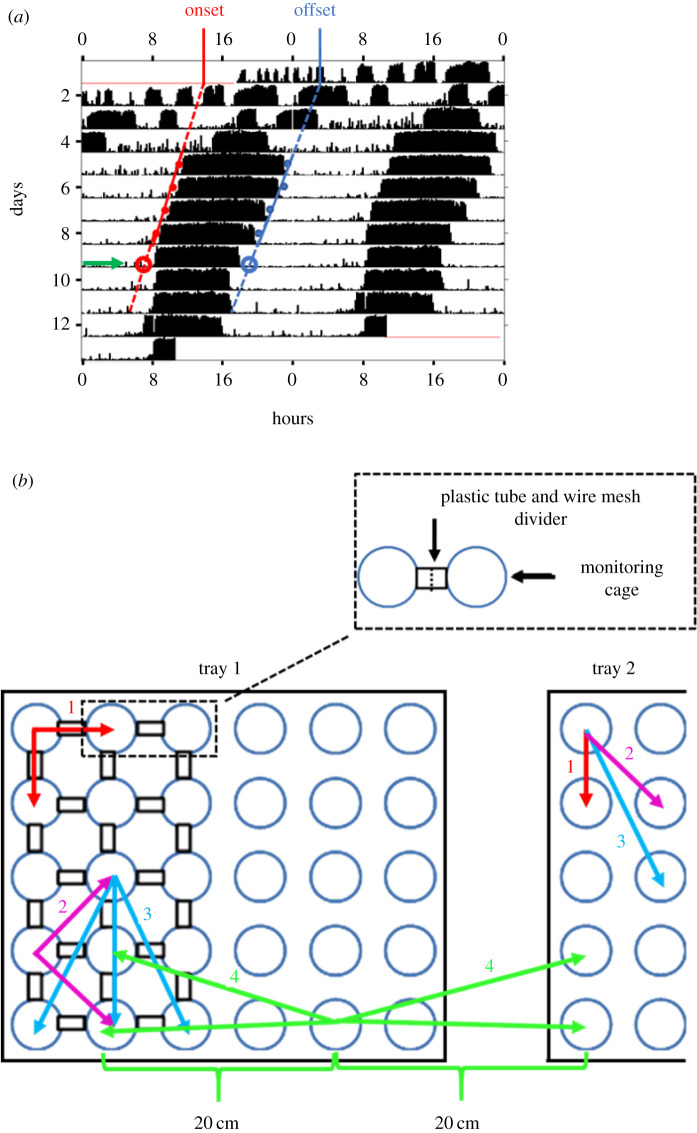
Methodological details. (*a*) Determining the phase of circadian rhythms in locomotor activity using a double-plot actogram. The *y*-axis shows days in the monitoring cage (which is also the age of the bee in this experiment), and the *x*-axis shows the time of day, double-plotted for easier visual detection of circadian rhythms. The height of the black bars within each day corresponds to the level of locomotor activity in a 10 min bin. The blue and red dots show the corrected software estimated times for the onset and offset of activity for each day, respectively. Linear regression models are fitted to these points and the phase is determined based on the extrapolation of the regression lines on day 9 (green arrow). Please note that young honeybee workers typically do not show circadian rhythms during the first day's post pupa eclosion. (*b*) Tray arrangement and summary of coupling strength analyses. A schematic illustration of one of the four trays in the monitoring chamber (part of a second tray is shown to the right). Each tray houses 30 cages that are constantly recorded by a video camera and a data acquisition system. Each tray is divided such that 15 cages are connected (left, the black rectangles mark transparent tubes in which we installed a wire mesh divider (dashed line) at approximately the middle of the tube) and 15 are not (right). The arrows and numbers depict the various types of coupling strength analyses we performed: (1) bees in cages connected or not connected to their direct neighbour cages. (2) Second-order neighbours. Connected or unconnected cages one step away from the direct neighbours. (3) Third-order neighbours. As in (2), but one step further away. (4) The influence of being placed on the same or on a different substrate. The coupling strength between bees in unconnected cages and similar bees on the same or on a different tray, but at a similar distance. (Online version in colour.)

We used the ClockLab circadian analyses software package (Actimetrics, USA) for the analyses of circadian rhythms. We used the *χ*^2^ periodogram analysis with 10 min bins to determine whether the activity rhythms of a given bee are statistically significant. As a proxy for the strength of circadian rhythms, we used the ‘*Power*’ which was calculated as the height of the periodogram plot peak above the *α* = 0.01 significance *p*-value threshold line (for more details see [79,80]). As indices for the phase, we recorded on each day the time of onset and offset of the daily bout of activity (honeybees are diurnal and typically show higher levels of activity during the day or subjective day). The precise time of the onset or offset was defined as at least three consecutive 10 min bins each with activity reaching at least 10% of the maximum activity per bin during this day and separated by a period of at least 5 h of reduced activity between the offset and the following onset (figure 1; following [27]). We used the ClockLab software package to fit linear regression models passing through the determined (as explained above) onset or the offset points of at least four consecutive days (figure 1, monitoring days 5–8) and used the extrapolations of these regression lines on the following day (day 9). This extrapolated time point is assumed to reflect the phase after sufficient time to permit social entrainment to a similar phase. We used for the analyses only bees with statistically significant circadian rhythms (*χ*^2^ periodogram analysis, *p* < 0.01; with a major period peak between 20–28 h) for which we could unambiguously determine the onset/offset of activity.

We used the Oriana circular statistics software package (KCS, USA) to determine the degree of synchronization and the phase coherence among bees within each treatment group using data of the bees for which we could unambiguously determine the phase. For all circular statistics analyses, we used the onset, offset and the median between these two indices as indices for phase. Given that for more bees, we could unambiguously determine the onset rather than the offset, and that the analyses using the three-phase indices were overall similar, we chose to present the onset data for which the statistical power is stronger. We used the Rayleigh test to determine if phase synchronization among a group of bees is significantly different from a random distribution. The mean length of the Rayleigh vector was used as an index for the degree of synchronization which in the framework of this experiment is an index for the degree of social synchronization.

### (d) Estimating the coupling function between bees

We used our customized data-driven graph-theoretic approach (ICON; [78]) to quantitatively describe the coupling function between each pair of bees. The pipeline we used includes a wide bandpass basic filter, the ICON calculation and connectivity analysis (see below), allowing us to efficiently and reliably infer the dynamic connectivity of oscillators from noisy measurements and is therefore appropriate for the locomotor activity data of honeybees. We modelled the honeybee locomotor activity data as a dynamic feature reflecting this complex dynamic network of honeybees with interactions, where the dynamics of each honeybee consists of its own rhythm and the influence from other honeybees. In particular, we consider the broadly defined complex network constituted by a population of *N* interacting honey bees (i.e. oscillators with a period to be 20–28 h). The time-evolution of such a network follows the dynamic law governed by the rhythm of honeybees *f*(*x_i_*) (i.e. oscillator's self-dynamics) and the influence by other honeybees *K_ij_*(*x_i_*, *x_j_*)(*x_i_*, *x_j_*), given by

2.1 $${\dot{x}}_{i}{\dot{x}}_{i}(t)=\;f(x_{i})+\overset{N}{\underset{\begin{array}{c} \;j=1\\ \;j\neq i \end{array}}{\sum }}K_{ij}(x_{i},x_{j});\;\;i=1,\ldots ,N,$$

where *x_i_*(*t*) is the locomotor activity of the *i*th honeybee at time *t*, the function *f*(*x_i_*) represents its baseline dynamics, such as its natural frequency, and *K_ij_*, *i*, *j* = 1, … ,*N*, is the coupling impact from the *j*th honeybee to the *i*th.

We first approximate the natural and coupling dynamics, *f* and *K_ij_* in equation (2.1), respectively, using complete orthonormal bases. Based on Kuramoto's model [81], we choose the Fourier base function with periods ranging from 16–32 h for our weakly coupled oscillatory honeybee network because *f* and *K_ij_* should be periodic functions (for more details see [78]). We next formulated this complex nonlinear estimation as a typical large-scale linear inverse problem for each honeybee:

2.2 $$\underset{z^{\left(i\right)}}{\min}∥\;y^{\left(i\right)}-A^{\left(i\right)}z^{\left(i\right)}{∥}_{2},$$

where $y^{\left(i\right)}\in R^{\left(M-1\right)}$ is the data vector whose elements $y_{j}^{\left(i\right)}=\Delta {\tilde{x}}_{j}^{\left(i\right)}/\Delta t_{j}$, *j* = 1, … , *M* − 1, denote the state difference with Δ*t_j_* = *t_j_*_+1_ − *t_j_* being the data sampling time interval; $A^{\left(i\right)}\in R^{\left(M-1)\times (2rN+1\right)}$ is the matrix involving orthonormal bases and *z*^(*i*)^ is the coefficient vector to be estimated which includes the connectivity information. The detailed formulation of equation (2.2) as well as the mathematical validation was as described with more details in [78]. A basic step for solving this large-scale linear inverse problem is to compute the Moore-Penrose pseudoinverse using the singular value decomposition (SVD). In this work, we examined the performances by implementing truncated singular value decomposition, which is more efficient compared to the standard SVD method because it only focuses on the most significant singular values that determine the linear inverse. Then, we can quantitatively measure the coupling strength from the *j*th honeybee to the *i*th (i.e. the magnitude of the function *K_ij_*) using the corresponding coefficients as in the solution *z*^(*i*)^. For the figure presentations, we normalized the data such that the maximal value was converted to 1, and the value of each measure was calculated as the proposition of this maximum giving a value ranging between 0 to 1.

### (e) The influence of direct contact on circadian rhythms in locomotor activity and phase synchronization

To test if cage connection improves social synchronization among newly emerged honeybee workers, we compared the locomotor activity rhythms of two groups of individually isolated worker bees. In the treatment group, adjacent cages were connected with transparent plastic tubes (length approximately 1.5 cm; inner diameter = 1 cm; figure 1*b* inset) with an 8′ wire mesh divider that was positioned in the middle of the tube. Thus, bees in connected cages could antennate and lick each other, but could not move from one cage to the other. The tube connection may also facilitate the propagation of substrate-borne vibrations and volatile chemicals. Bees of the control treatment were similar and were housed in identical cages and distance from each other, but the cages were not connected and the bees could not contact their neighbours (figure 1*b*). We monitored and analysed locomotor activity, circadian rhythms and circular statistics as described above. We performed separate analyses for each treatment group on a tray (i.e. ‘connected’ or ‘not connected’; *n* = 15 bees; figure 1*b*). We calculated the percentage of bees that survived until the end of the experiment and the percentage of bees with significant circadian rhythms in locomotor activity. We then used Pearson chi-square tests to assess the effect of cage connection on these two variables, and two-way-ANOVA to analyse differences in strength (power of rhythmicity) of circadian rhythms. We repeated this experiment three times monitoring locomotor activity for a total of 261 newly emerged worker bees. For the circadian analysis, we used 198 bees for which we could unambiguously detect the onset of the daily bout of activity (69, 57 and 63, in trials 1, 2 and 3, respectively).

### (f) The influence of direct contact, distance and being on the same substrate on coupling strength

We used the ICON pipeline (see above) to calculate the coupling function for each pair of focal bees (‘oscillators’) allowing us to precisely compare the influence of direct contact, distance on the tray, and the effect of whether the bees were placed on the same or on a different tray. The coloured arrows and numbers in figure 1*b* summarize our different analyses: (i) bees with and without direct contact with their neighbour bees (direct neighbours); (ii) second-order neighbours, who are in cages one step away from the direct neighbours (figure 1*b*); (iii) third-order neighbours (as in (ii), but one step further away); and (iv) unconnected bees to others on the same or on a different tray, but at a similar distance. This analysis can separate olfactory from vibratory synchronization because the former, but not the latter is expected to be equal for bees at a similar distance on the same versus on a different tray (i.e. substrate). For the ICON analysis, we used 183 bees. This number is lower than for the circadian analyses because for robust ICON analyses, we include only bees for which we had good locomotor activity records for at least 10 successive days (77, 51 and 55, in trials 1, 2 and 3, respectively).

## 3. Results

**Figure 2.**
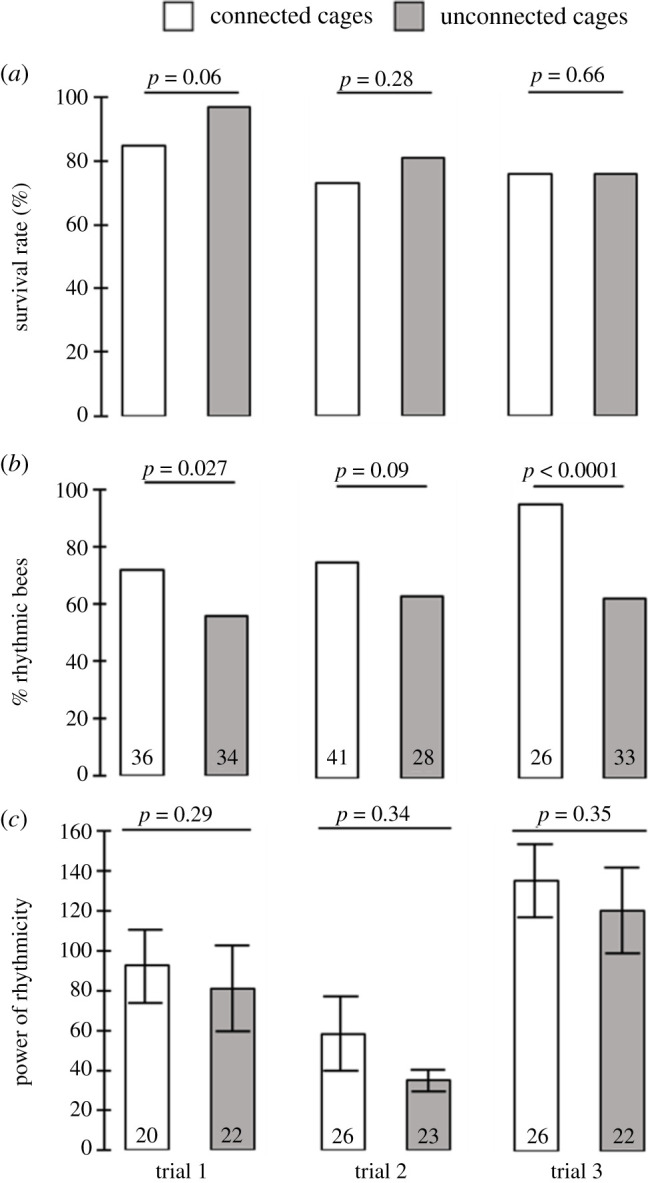
The influence of cage connection on survival and 24 h rhythms in locomotor activity. (*a*) Percentage of bees that survived until the end of the monitoring session. The *p*-values above the plots summarize the results of Pearson chi-square tests. (*b*) Percent rhythmic bees out of the ones survived until the end of the monitoring session (sample size within bars, other details as in (*a*)). (*c*) The ‘power’ as an index for the strength of circadian rhythms in locomotor activity. The influence of treatment was not significant in a two-way-ANOVA (see text for details). The plots show mean ± s.e., with the sample size within bars.

Survival rate was similar for bees in connected and unconnected cages (trial 1: 85% versus 97%; trial 2: 73% versus 81%; trial 3: 76% versus 76%, respectively; Pearson chi-square test *p* = 0.06, *p* = 0.28, *p* = 0.66 for trials 1, 2 and 3, respectively; figure 2*a*).

### (a) The influence of direct contact on circadian rhythms in locomotor activity and phase synchronization

**Figure 3.**
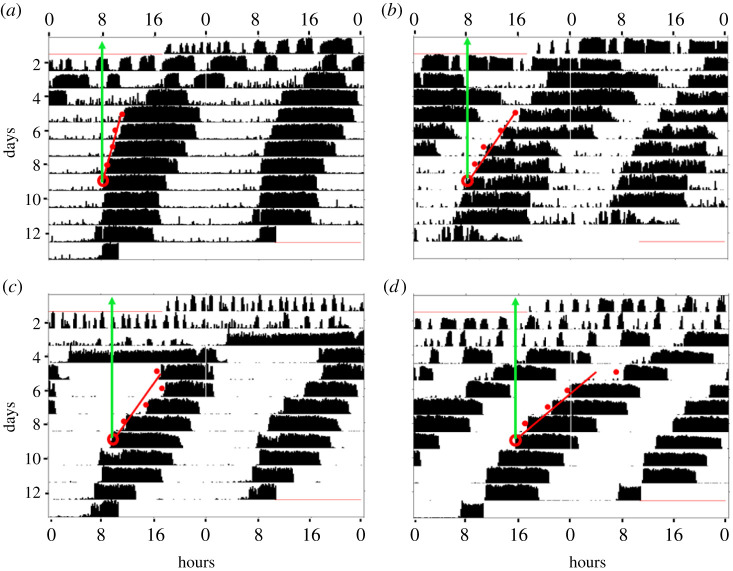
Representative actograms showing the locomotor activity of individually isolated honeybee workers placed on the same tray. (*a*,*c*) Actograms of two individuals housed in cages connected to each other. (*b*,*d*) Same as above but in a part of the tray in which the cages were not connected to each other. Details of actograms as in figure 1. The red dots show the estimated onset of the daily bout of activity, and the red line is the best linear regression model fitting these dots. The open circles depict the extrapolated onsets on day 9 and the green arrow aims to facilitate precise reading of the estimated time of onset. (Online version in colour.)

Bees in connected cages were more likely to show statistically significant approximately 24 h rhythms in locomotor activity (figure 2*b*; Pearson chi-square test *p* = 0.027, *p* = 0.09, *p* < 0.0001, for trials 1, 2 and 3, respectively). When looking only at the bees that developed statistically significant rhythms, there was no difference in the strength of approximately 24 h rhythms between bees in connected (mean ± s.e. = 92.4 ± 21.1, 58.3 ± 20.6, 135.3 ± 23.2) and unconnected cages (80.9 ± 27.1, 34.7 ± 6, 120.1 ± 25.1, for trials 1, 2 and 3, respectively; two-way-ANOVA, treatment – *F* = 1.13, *p* = 0.29; trial – *F* = 7.29, *p* = 0.001; treatment × trial, *F* = 0.041, *p* = 0.96; figure 2*c*). Figure 3 presents representative actograms of bees from cages placed on the same tray. The actograms show that the extrapolated onset on day 9 of the experiment is more similar for the bees in connected compared to non-connected cages.

**Figure 4.**
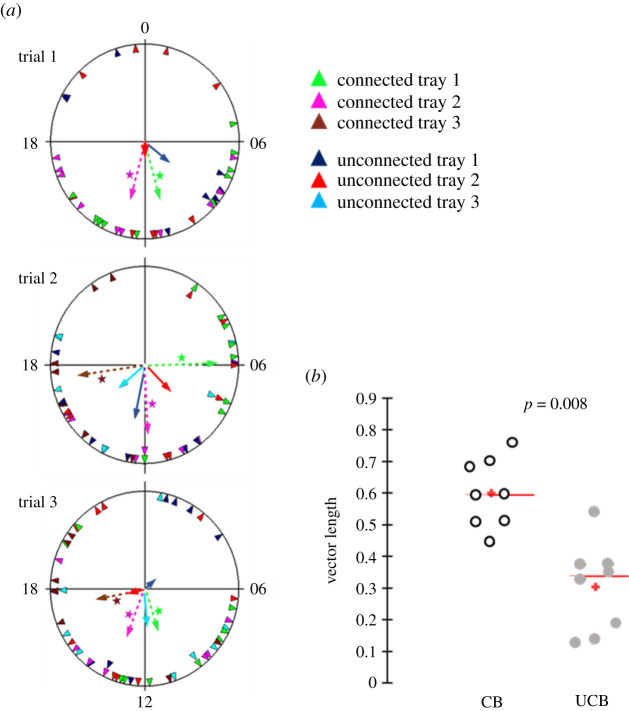
The influence of cage connection on social synchronization. (*a*) Circular statistics. The time of day is depicted on the plot perimeter. The onset of the daily bout of activity was used as an index for the phase of circadian rhythms in locomotor activity; each triangle depicts the onset of an individual bee. The vectors point to the average onset time (mean vector), and their length corresponds to the degree of synchronization. Asterisks indicate that the phases of bees in the same groups are significantly different from random distribution in a Rayleigh test (*−*α* < 0.05). Each plot summarizes the results of a different trial. Dashed lines, connected cages; continuous lines, unconnected cages. (*b*) A scatter plot summary of the degree of synchronization for all three trials. The red crosses correspond to the means; the central horizontal bars are the medians. CB, bees in connected cages, UCB, bees in unconnected cages. The *p*-value on the top summarizes the result of a Wilcoxon signed-rank test (based on *n* = 8 Rayleigh vectors). (Online version in colour.)

In 5 out of 8 trays, the bees in connected cages had stronger phase coherence (longer Rayleigh vector; Rayleigh test: *p* = 0.017, *p* = 0.023, *p* = 0.006, *p* = 0.032, *p* = 0.015, *p* = 0.117, *p* = 0.12, *p* = 0.16; figure 4*a*) compared to bees on the same tray but housed in unconnected cages (Rayleigh test: *p* = 0.39, *p* = 0.87, *p* = 0.13, *p* = 0.33, *p* = 0.43, *p* = 0.88, *p* = 0.74, *p* = 0.33; figure 4*a*). In a pooled analysis across the three trials, the length of the Rayleigh vector was significantly longer (better synchronization) for the bees in connected cages (figure 4*b*; Wilcoxon signed-rank test; *p* = 0.008, *n* = 8).

### (b) The influence of direct contact, distance and being on the same substrate on coupling strength

**Figure 5.**
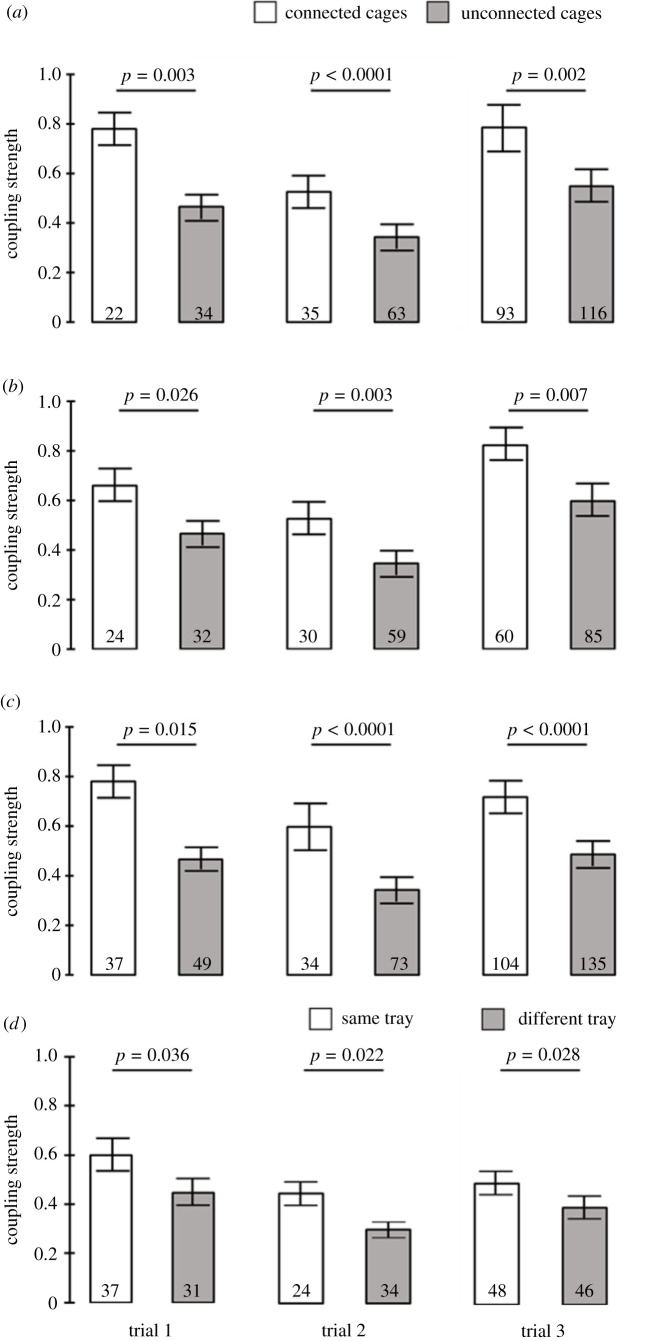
The influence of cage connection and being on the same substrate on coupling strength. (*a*) Bees in adjacent cages; (*b*) second-order neighbours; (*c*) third-order neighbours. (*d*) Bees in unconnected cages at a similar distance but placed on the same or on a different tray (figure 1*b* for details). The bars show mean ± s.e., sample size is shown within bars. The *p*-values above the bars summarize the results of unpaired *t*-tests.

In the first analysis, we compared the coupling strength for pairs of bees in adjacent cages with or without tube connection (no. 1 in figure 1*b*). We found that cage connection augmented the coupling strength in all three trials (*t*-test; *p* = 0.003; *p* < 0.0001; *p* = 0.002, in trials 1, 2 and 3, respectively; figure 5*a*). The array of connected cages also improved coupling strength in all three trials when we compared second-order neighbours (no. 2 in figure 1*b*; *p* = 0.026; *p* = 0.003; *p* = 0.007, in trials 1, 2 and 3, respectively; figure 5*b*). A similar effect of cage connection was also found in all three trials for third-order neighbours (no. 3 in figure 1*b*) that are further apart from each other (*p* = 0.015; *p* < 0.0001; *p* < 0.0001, respectively; figure 5*c*). Finally, we compared the coupling strength of bees in unconnected cages at a similar distance but placed either on the same or on a different tray (no. 4 in figure 1*b*). We found that bees on the same tray have significantly higher coupling strength values compared to bees at a similar distance but caged on a different tray (*p* = 0.036; *p* = 0.022; *p* = 0.028, respectively; figure 5*d*).

## 4. Discussion

Honeybees live in populated nests in which their activities interact to influence the hive social and physical environment. Studies reviewed above show that environmentally entrained forager activity entrains the circadian clocks of nest bees. The nest bees are synchronized with each other and with ambient day-night cycles even if prevented from sampling the environment outside the hive or in the hive periphery. This social entrainment does not require direct contact between the nest bees and the foragers [27,55,67]. The results of the experiments reported here show that social synchronization is also effective in groups composed of only callow bees which typically have attenuated circadian rhythms (weak oscillators). Moreover, we show that callow bees in individual cages which are connected by a small tube are more likely to show circadian rhythms in locomotor activity, are better synchronized with each other and have stronger coupling strength, compared to similar bees placed in similar cages that were not connected to each other. Given that only about 100 bees were placed in an entire environmental chamber, it is unlikely that they could effectively regulate the chamber physical environment (e.g. temperature, humidity). The bees were spread apart and not crowded as in typical colonies or in previous laboratory experiments, further obstructing their competence to create a common microenvironment. These new findings extend our understanding of social synchronization in honeybees by showing that social cues much weaker than appreciated before can synchronize the daily activity of bees to a common phase. These results are robust because they are based on three trials, each with bees from a different source colony and a large dataset of more than 250 individual bees. Given that bees in each colony are the offspring of a different queen and drones, our findings are not limited to certain genotypes or laboratory lines.

The bees in connected cages were better synchronized with each other compared to similar bees in unconnected cages showing that the tube connecting the cages facilitated phase synchronization. The coupling strength analyses are consistent with the circular statistics, by showing higher values for bees in connected cages even when we compared second- and third-order neighbours (figure 5). At least two social mechanisms can account for these findings. First, direct contact by means of tactile or chemical communication via the mesh separation improved the synchronization of each pair of neighbours to a common phase. Given that all the bees were connected in an array of cages, the information could eventually spread to the network and entrain the bees to a similar phase. Social synchronization according to this scenario can be analysed using social network models. Second, social synchronization is not mediated by direct contact but by creating a common environment inside the array of connected cages. According to this explanation, the tubes connecting the cages improved the propagation of surrogates of activity such as substrate-borne vibrations or volatiles [48,67,82] creating a common oscillating microenvironment which is composed of only the connected cages and tubes. These oscillations in the common environment in turn affect the activity of each bee, and eventually all the bees are synchronized to a similar phase. Additional studies are necessary for distinguishing between these two hypothesized social synchronization mechanisms.

The bees we studied here were collected shortly after emerging from the pupa and were monitored at a young age in which circadian and daily rhythms are typically weak or absent, but later develop robust circadian rhythms (reviewed in [17,57,83]). The development of circadian rhythms is socially regulated with callow bees placed together with foragers showing stronger rhythms and faster rhythm development compared to same-age sister bees that were housed with a similar number of young bees [27,28,55,77]. Previous studies showed that social synchronization can also be achieved in a group of callow bees in a cage, but synchronization is weaker compared to bees experiencing the hive environment [27]. Given their weak rhythms and propensity to be entrained by older bees, it is remarkable that we found that a relatively small number of dispersed callow bees with limited contact with other callow bees nevertheless were able to achieve efficient phase synchronization. These findings suggest that honeybees are extremely sensitive to the social cues mediating social synchronization, and that this sensitivity has already developed at a young age. Consistent with this premise, Fuchikawa *et al*. [27] showed that exposure to the colony environment during the first 2 days (but not a single day) post-pupal eclosion is sufficient to entrain callow bees to the colony phase.

**Figure 6.**
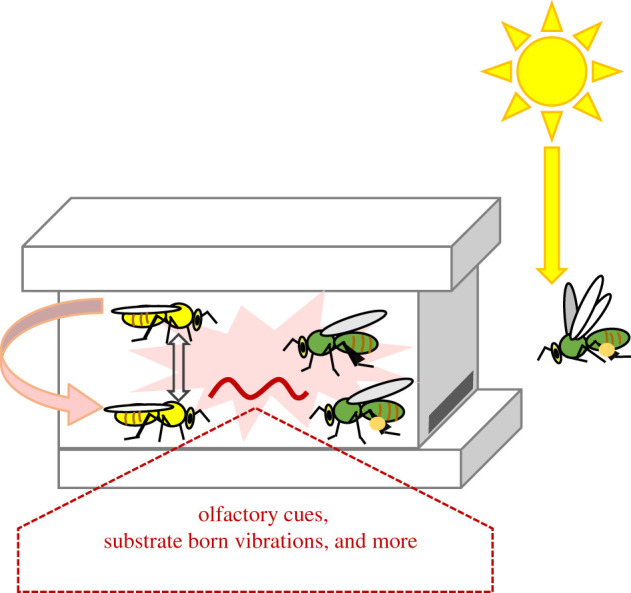
A schematic self-organized model for social synchronization of circadian rhythms in honeybee colonies. Foragers (painted green) are entrained by ambient day-night cycles and show higher activity during the day. Surrogates of forager activity in the hive such as olfactory cues or comb vibrations create oscillations in the common nest environment (peach colour background). These oscillations (red cosine wave) in turn entrain a growing number of bees to the same phase. The more bees that are synchronized to the same phase, the stronger the oscillations in the nest environment and their competence to entrain circadian rhythms in additional bees. The peach colour arrow to the left shows that young bees (painted yellow) can also mutually synchronize each other to a common phase by similar mechanisms. The double-head open arrows refer to new results presented here which may suggest that direct contact improves social entrainment among pairs of young bees. It is still unknown whether close-distance contact also improves synchronization among older bees (such as foragers) and whether direct contact is sufficient for social entrainment of circadian rhythms. (Online version in colour.)

What are the social cues for which the callow bees are so sensitive? The power of the ICON pipeline enabled us to identify one such cue: substrate-borne vibrations. The findings that bees on the same substrate (tray) showed stronger coupling strength compared to bees at the same distance but caged on a different substrate (figure 5*d*) are consistent with synchronization mediated by substrate-borne vibrations. By contrast to substrate-borne vibrations, volatile chemical cues and airborne vibrations (e.g. auditory cues) are expected to be affected by distance but not by substrate sharing. Thus, even the relatively weak vibrations generated by the activity of a small number of bees on the same tray were sufficient for social synchronization, resembling in a sense the famous metronome synchronization experiments [84]. Bees detect air movements and substrate vibrations via sensory organs in their antennae and legs and use vibratory signals for communication (reviewed in [75]). The premise that vibrations can entrain circadian rhythms is supported by experiments in which vibratory signals entrained circadian rhythms in the fruit fly *D. melanogaster* [76]. The studies with the fruit fly indicate that vibratory information from mechanosensory receptors can reach and entrain cells of the circadian network controlling locomotor activity. The current findings support and extend our previous study in which we showed that young bees are entrained to the activity phase of foragers placed in a cage on the same substrate, but not at a similar distance but on a different substrate [67]. Additional social cues to which callow bees may be very sensitive are volatile chemicals. For example, Moritz & Kryger [48] showed that opening small holes in the wall separating two groups of bees improved social synchronization, and we previously showed that air drawn from a free-foraging colony can entrain young bees to the colony phase [67]. Figure 6 summarizes our current understanding of social synchronization in honeybee colonies integrating information reviewed in the first part of this paper and the new experiment presented in the second part.

In a broader view, the current study with honeybees supports earlier studies suggesting that complex group behaviour such as crickets that chirped in unison, the concerted action of shimmering waves in *Apis dorsata* and synchronously flashing fireflies can be explained by coupled oscillator theory approaches [85]. Actually, there is evidence that collective behaviours are similar across levels of biological organizations ranging from small groups of 2 or 5 mice, to hundreds of cells in the suprachiasmatic nucleus [78]. The dynamics of these systems, as well as this of coupled chemical oscillators, can be described by extensions of Kuramoto's phase model, a mathematical model to describe behaviour (e.g. synchronization) of coupled oscillators [81]. The new results presented in this paper show that that this approach can be extended to noisy locomotor activity data, and for general networks of weak oscillators.

## Data accessibility

The detailed development of the ICON equations are reported in [78] which is cited in our paper.
